# Association of *ABCG2* rs2231142 Allele and BMI With Hyperuricemia in an East Asian Population

**DOI:** 10.3389/fgene.2021.709887

**Published:** 2021-08-31

**Authors:** Yen-Ju Chen, I-Chieh Chen, Hsueh-Ju Lin, Ying-Cheng Lin, Jui-Chun Chang, Yi-Ming Chen, Tzu-Hung Hsiao, Pei-Chun Chen, Ching-Heng Lin

**Affiliations:** ^1^Department of Medical Research, Taichung Veterans General Hospital, Taichung, Taiwan; ^2^Division of Allergy, Immunology and Rheumatology, Department of Internal Medicine, Taichung Veterans General Hospital, Taichung, Taiwan; ^3^Institute of Clinical Medicine, National Yang Ming Chiao Tung University, Taipei, Taiwan; ^4^Division of Gastroenterology and Hepatology, Department of Internal Medicine, Taichung Veterans General Hospital, Taichung, Taiwan; ^5^Department of Obstetrics and Gynecology and Women’s Health, Taichung Veterans General Hospital, Taichung, Taiwan; ^6^Institute of Biomedical Science and Rong Hsing Research Center for Translational Medicine, National Chung Hsing University, Taichung, Taiwan; ^7^School of Medicine, National Yang Ming Chiao Tung University, Taipei, Taiwan; ^8^Department of Public Health, College of Medicine, Fu Jen Catholic University, New Taipei City, Taiwan; ^9^Institute of Genomics and Bioinformatics, National Chung Hsing University, Taichung, Taiwan; ^10^Department of Mathematics and Information Education, National Taipei University of Education, Taipei, Taiwan; ^11^Department of Health Care Management, National Taipei University of Nursing and Health Sciences, Taipei, Taiwan; ^12^Department of Industrial Engineering and Enterprise Information, Tunghai University, Taichung, Taiwan; ^13^Institute of Public Health and Community Medicine Research Center, National Yang Ming Chiao Tung University, Taipei, Taiwan

**Keywords:** uric acid, hyperuricemia, gout, *ABCG2* rs2231142, BMI, obesity

## Abstract

**Objectives:** Genetic variants and obesity are risk factors for hyperuricemia (HUA). Recent genome-wide association studies have identified *ABCG2* rs2231142 as one of the most prominent genetic variants for HUA in an East Asian population. Nevertheless, no large-scale studies have demonstrated any interactive effects between this variant and obesity on serum urate level in Asians. This study aimed to determine the interaction of *ABCG2* rs2231142 variant and body mass index (BMI) and its effect on risk of HUA in an East Asian population.

**Methods:** The study was conducted using the Taiwan Biobank database, a population-based biomedical research database of patients with Taiwanese Han Chinese ancestry aged 30–70years between September 2014 and May 2017. Detailed physical information on participants were collected by questionnaires and genotyping using Affymetrix TWB 650K SNP chip. The primary outcome was HUA, defined as a serum uric acid level>7.0mg/dl. Odds ratio (OR) of HUA was analyzed using logistic regression models and the effects of interaction between *ABCG2* rs2231142 variants and BMI on serum uric acid level were explored.

**Results:** We identified 25,245 subjects, 4,228 (16.75%) of whom had HUA. The prevalence of HUA was 30% in men and 3.8% in women. The risk of HUA was significantly associated with *ABCG2* rs2231142 risk T allele, with more HUA in TT genotype (OR: 2.40, 95% CI: 2.11–2.72, *p*<0.001) and TG genotype (OR: 1.64, 95% CI: 1.51–1.78, *p*<0.001) in men, and TT genotype (OR: 2.42, 95% CI: 1.83–3.20, *p*<0.001) and TG genotype (OR: 1.82, 95% CI: 1.46–2.23, *p*<0.001) in women, compared with their counterparts. Moreover, we found a strong genetic-environmental interaction associated with the risk of HUA. There was increased risk of HUA by the interaction of *ABCG2* rs2231142 variant and BMI for TT genotype (OR: 7.42, 95% CI: 2.54–21.7, *p*<0.001) and TG genotype (OR: 4.25, 95% CI: 2.13–8.47, *p*<0.001) in men compared with the GG genotype in men, and for TT genotype (OR: 25.43, 95% CI: 3.75–172.41, *p*<0.001) and TG genotype (OR: 3.05, 95% CI: 0.79–11.71, *p*=0.011) in women compared with the GG genotype in women.

**Conclusion:** The risk of HUA was markedly increased by the interaction of *ABCG2* rs2231142 variant and BMI, both in men and in women. Body weight control and reduction in BMI are recommended in high-risk patients with the *ABCG2* rs2231142 risk T allele.

## Introduction

Hyperuricemia (HUA; serum uric acid levels>7.0mg/dl) is a major risk factor for gout, which is known as “the disease of kings and the king of diseases.” Acute gouty attacks cause severe pain, difficulty in walking, functional impairments, and major disability, as well as decreased productivity if left untreated. Moreover, HUA is associated with multiple comorbidities, including obesity, hypertension, type 2 diabetes, hyperlipidemia, cardiovascular diseases, chronic kidney diseases, stroke, osteoporosis, erectile dysfunction, obstructive sleep apnea, and is an independent predictor of premature mortality (Vincent et al., 2016; Singh and Cleveland, 2019; Huang et al., 2020). The treatment target of gout is to reduce the levels of serum uric acid, and then to minimize medical comorbidities.

A number of genes associated with gout and HUA were identified by genome-wide association studies (Köttgen et al., 2013; Liu et al., 2020), and most were related to the urate-transport system, such as ATP-binding cassette subfamily G member 2 (*ABCG2*), glucose transporter type 9 (*GLUT9*, also known as *SLC2A9*), and urate anion transporter 1 (*URAT1*, also known as *SLC22A12*). *ABCG2* encodes a high-capacity transporter for urate efflux and the *ABCG2* rs2231142 single nucleotide polymorphism (SNP) is one of the most prominent genetic variants associated with HUA and gout (Dehghan et al., 2008; Woodward et al., 2009). Moreover, the association between *ABCG2* rs2231142, HUA and gout risk varies with ethnicity (Dong et al., 2015), with an almost 3-fold increase of T-risk allele frequency in the East Asian population compared to the European population (Okada et al., 2012).

Obesity is one of the major risk factors for HUA, and body mass index (BMI) was positively correlated with serum uric acid levels in previous studies (R=0.96; *p*<0.00001; Shirasawa et al., 2020; Yu et al., 2021). Tai et al. (2020) has reported increased risk of gout when urate genetic risk scores are higher in low/normal-weight (OR: 2.89, 95% CI: 2.42–3.47), overweight (OR 3.09, 95% CI: 2.84–3.36), and obese individuals (OR 2.65, 95% CI: 2.46–2.86) based on BMI. Cheng et al. (2017) have reported that interaction effects between *ABCG2* rs2231142 and obesity on serum uric acid levels in women in a Han Taiwanese population (*p* for interaction=0.0189). Other studies have also demonstrated that interactions between BMI and genetic variants affect serum urate concentrations and gout risk, but with conflicting results (Brandstätter et al., 2008; Li et al., 2013). However, it may not be possible to use the previously developed urate genetic risk score for non-European ancestry. Also, the score includes some SNPs with a very low minor allele frequency, which might reduce the power (Tai et al., 2020). Furthermore, previous studies using single SNP analysis revealed conflicting results, which were likely due to the fact that a single ethnicity was studied and the sample sizes were relatively small. In addition to the sample size (*n*=459), the definition of obesity was obscure in a study of *ABCG2* rs2231142 in East Asians (Cheng et al., 2017). Therefore, it is currently unclear whether *ABCG2* rs2231142 contributes to serum uric acid level according to BMI in East Asians. This study aimed to determine the impact of the interaction between *ABCG2* rs2231142 and BMI on serum uric acid level, according to gender, in an East Asian population using a large sample size.

## Materials and Methods

### Participants

This study was conducted using the Taiwan Biobank (TWB), a population-based biomedical research database of patients with Taiwanese Han Chinese ancestry aged 30–70years, without a history of cancer. The patients were enrolled from 29 recruitment centers throughout Taiwan and all of the participants provided informed consent. Our study cohort was composed of 25,245 individuals with genotyping information, serum uric acid reports and the demographics, medical history, lifestyle modality, body fat evaluation including BMI, waist circumference, and body fat percentage, as well as biochemical reports including serum creatinine, cholesterol and fasting glucose level, were all identified from the database. The study was conducted in accordance with the Declaration of Helsinki and was approved by the ethics committee of Taichung Veterans General Hospital’s Institutional Review Board (IRB no. CE16270B-2). All available data were achieved from TWB, which collected specimens and information in a complete and standardized procedure to fit researchers’ needs in different fields (Pan et al., 2006; Chen et al., 2016; Lin et al., 2019).

### Genotyping and Quality Controls

The individual genotyping information was obtained from the TWB participants. A qualified and trained researcher with a medical background assisted in collecting 30-ml blood and extracting DNA from blood sample by employing QIAamp DNA blood kits following the manufacturer’s instructions (Qiagen, Valencia, CA, United States). After evaluating the quality of the isolated genomic DNA (Pan et al., 2006; Chen et al., 2016), it was genotyped by using custom Taiwan BioBank 2.0 SNP chip and an Axiom Genome-Wide Array Plate System (Affymetrix, Santa Clara, CA, USA) at the National Center for Genome Medicine in Academia Sinica, Taiwan. Affymetrix TWB 2.0 SNP chip contained 653, 291 SNPs and was designed specifically for Taiwan’s Han Chinese population. The genotype information and linkage disequilibrium of participants were released by the Ethics and Governance Committee (EGC) of Taiwan Biobank (TaiwanView: https://taiwanview.twbiobank.org.tw/index; Fan et al., 2008). PLINK was used for analysis and a quality control procedure was performed to exclude markers that failed Hardy–Weinberg equilibrium tests with value of *p*<1×10^−6^, minor allele frequency<0.01, and a genotyping call rate less than 90% (Purcell et al., 2007).

### Data Collection and Outcome Identification

Body fat evaluation including BMI, waist circumference, waist-hip ratio, and body fat percentage were obtained. BMI was weight in kilograms divided by height in meters squared (kg/m^2^) and overweight was defined as BMI≥24kg/m^2^ (for the East Asian population). Body fat percentage was the percentage of fat by weight (Kelishadi et al., 2015). Moreover, baseline characteristics of social history (habits of alcohol drinking, smoking, and betel nut chewing) and physical activity were collected by a questionnaire through a face-to-face interview with one of the Taiwan Biobank researchers. Alcohol use was defined as a weekly intake of more than 150ml alcohol for at least 6months and smoking was defined as daily use of tobacco for an uninterrupted period of at least 6months. The social history status was dichotomized as a current- or ever-user vs. a non-user. The extent of physical activity was dichotomized as non-regular vs. regular exercise, which was defined as exercise for more than 30min at least three times a week. Biochemical data including serum creatinine, lipid profile, and fasting glucose level were also collected and provided by the TWB database. Hyperlipidemia was defined as a level of total cholesterol ≥200mg/dl, low density lipoprotein (LDL)≥100mg/dl, triglyceride (TG)≥150mg/dl, or high density lipoprotein (HDL)<40mg/dl in men or <50mg/dl in women. Hyperglycemia was defined as fasting glucose level≥100mg/dl.

The primary outcome was hyperuricemia, which was defined as a level of serum uric acid>7.0mg/dl. The level of serum uric acid was measured using an Architect i2000SR Analyzer (Abbott Diagnostics, Abbott Park, Chicago, IL, United States) by the uricase method (Liao et al., 2006).

### Statistical Analysis

The demographic information is shown as mean±standard deviation for continuous variables and number (percent) for categorical variables. Student’s *t*-test for continuous variables and Chi-square test for categorical variables were used to compare data between HUA and non-HUA controls. Odds ratios (OR) and 95% confidence interval (95% CI) of variables and *ABCG2* rs2231142 on HUA were calculated by logistic regression. Multiple logistic regression models were also used for evaluating the effect of interaction of *ABCG2* rs2231142 and BMI on HUA. All data were analyzed using R, version 4.0.0 software. Statistically significance was set at *p* values less than 0.05.

## Results

### Baseline Characteristics of the Participants

Between September 2014 and May 2017, we identified 25,245 subjects in the final analysis, including 4,228 (16.75%) with hyperuricemia and 21,017 (83.25%) in the control group (Table 1). The prevalence of HUA was 30% in men and 3.8% in women. More postmenopausal women aged 55–70years had HUA compared to the control group (54 vs. 29%, *p*<0.001) but surprisingly, more men aged 30–45years had HUA (48%). Higher BMI (men: 26.57±3.51 vs. 24.69±3.34kg/m^2^, *p*<0.001; women: 27.20±4.39 vs. 23.37±3.60kg/m^2^, *p*<0.001), waist circumference (men: 90.79±9.21 vs. 86.25±9.03cm, *p*<0.001; women: 89.86±10.46 vs. 70.14±9.55cm, *p*<0.001), waist-hip ratio (men: 0.91±0.05 vs. 0.89±0.06, *p*<0.001; women: 0.90±0.07 vs. 0.84±0.07, *p*<0.001), and body fat percentage (men: 24.80±5.18 vs. 21.99±5.36, *p*<0.001; women: 37.63±6.91 vs. 31.38±6.30, *p*<0.001) were observed in both men and women with HUA, compared with their counterparts. Furthermore, those with HUA were more likely to be users of alcohol or betel nut, and exhibited higher systolic blood pressure, higher blood total cholesterol level, higher LDL level, higher triglyceride level, but lower HDL level, compared with those without HUA.

**Table 1:** Baseline characteristics of the participants.

Variables	Male (*N*=12,440)			Female (*N*=12,805)		
	Without HUA (*n*=8,699)	With HUA (*n*=3,741)	Sig.	Without HUA (*n*=12,318)	With HUA (*n*=487)	Sig.
Age (years; %)^a^						
30–45	3,539 (41)	1786 (48)		5,371 (44)	88 (18)	
45–55	2,268 (26)	958 (26)		3,320 (27)	136 (28)	
55–70	2,892 (33)	997 (27)	***	3,627 (29)	263 (54)	***
Alcohol consumption (%)^a^						
No	7,704 (89)	3,142 (84)		12,102 (98.28)	470 (96.51)	
Yes	988 (11)	596 (16)	***	212 (1.72)	17 (3.49)	**
Smoking (%)^a^						
No	3,903 (45)	1,591 (43)		11,087 (90.04)	441 (90.55)	
Yes	4,784 (55)	2,142 (57)	*	1,227 (9.96)	46 (9.45)	
Betel nut chewing (%)^a^						
No	7,460 (86)	3,091 (83)		12,283 (99.78)	483 (99.18)	
Yes	1,233 (14)	646 (17)	***	27 (0.22)	4 (0.82)	*
Physical activity (%)^a^						
No	4,925 (57)	2,285 (61)		7,477 (60.74)	276 (56.67)	
Yes	3,770 (43)	1,453 (39)	***	4,832 (39.26)	211 (43.33)	
Body mass index (kg/m^2^)^b^	24.69±3.34	26.57±3.51	***	23.37±3.60	27.20±4.39	***
Waist circumference (cm)^b^	86.25±9.03	90.79±9.21	***	70.14±9.55	89.86±10.46	***
Waist-hip ratio^b^	0.89±0.06	0.91±0.05	***	0.84±0.07	0.90±0.07	***
Body fat percentage^b^	21.99±5.36	24.80±5.18	***	31.38±6.30	37.63±6.91	***
Blood pressure (mmHg)^b^	124.86±17.04	127.81±17.25	***	115.77±18.37	129.37±18.96	***
Total cholesterol (mg/dl)^b^	189.06±33.92	195.74±36.52	***	195.07±35.52	204.97±40.76	***
Triglyceride (mg/dl)^b^	122.19±92.89	164.41±134.94	***	98.54±88.66	150.16±91.50	***
HDL cholesterol (mg /dl)^b^	49.05±11.27	45.01±10.06	***	58.18±13.14	49.84±11.10	***
LDL cholesterol (mg/dl)^b^	119.63±30.62	124.39±32.60	***	118.46±31.26	129.24±36.93	***
Fasting glucose (mg/dl)^b^	99.46±26.14	97.93±17.77	***	93.10±17.57	101.78±22.63	***

HUA, hyperuricemia (defined as serum uric acid level>7.0mg/dl); Sig., significant; HDL, high density lipoprotein; LDL, low density lipoprotein.

a Categorical variables were expressed as numbers (percent) and were analyzed using the Chi-square test.

b Continuous variables were expressed as mean±standard deviation (SD) and were analyzed using Student’s *t*-test for normal data distributions.

* :statistically significant at 0.01<*p*≤0.05;

** : 0.001<*p*≤0.01;

*** *: p*≤0.001.

### Genotype Frequencies of *ABCG2* rs2231142 and Association With HUA

The genotype frequency of *ABCG2* rs2231142 is shown in Table 2. More TT and TG genotypes of *ABCG2* rs2231142 were found in the HUA group than in the control group, with a similar distribution in men (TT: 14.5 vs. 8.3%, *p*<0.001; TG: 48.8 vs. 41.2%, *p*<0.001) and women (TT: 15.9 vs. 9.8%, *p*<0.001; TG: 52.2 vs. 42.7%, *p*<0.001). The minor T allele of *ABCG2* rs2231142 was 38.9% in the HUA population, compared with 28.9% in the control group in men, and 42.0 and 31.1% in females, respectively. Furthermore, the risk of HUA was significantly associated with *ABCG2* rs2231142 risk T allele, and both TT and TG genotypes contributed to increased risk of HUA in men (TT: OR: 2.40, 95% CI: 2.11–2.72, *p*<0.001; TG: OR: 1.64, 95% CI: 1.51–1.78, *p*<0.001) and women (TT: OR: 2.42, 95% CI: 1.83–3.20, *p*<0.001; TG: OR: 1.82, 95% CI: 1.46–2.23, *p*<0.001) compared with their counterparts.

**Table 2:** Genotype and allele frequencies of *ABCG2* rs2231142 and risk of hyperuricemia in the participants.

Gene/SNP	Male (*N*=12,440)				Female (*N*=12,805)			
	Without HUA (*n*=8,699)	With HUA (*n*=3,741)	OR (95% CI)	*p*	Without HUA (*n*=12,318)	With HUA (*n*=487)	OR (95% CI)	*p*
*ABCG2* rs2231142								
GG	4,389 (50.5)	1,369 (36.6)	1		5,851 (47.6)	155 (32.0)	1	
TG	3,580 (41.2)	1827 (48.8)	1.64 (1.51, 1.78)	<0.001	5,245 (42.7)	253 (52.2)	1.82 (1.46, 2.23)	<0.001
TT	724 (8.3)	541 (14.5)	2.40 (2.11,2.72)	<0.001	1,202 (9.8)	77 (15.9)	2.42 (1.83, 3.20)	<0.001
Allele								
G	17,386 (71.1)	4,565 (61.1)			16,947 (68.9)	563 (58.0)		
T	5,028 (28.9)	2,909 (38.9)	2.20 (2.08,2.33)	<0.001	7,649 (31.1)	407 (42.0)	1.60 (1.41,1.82)	<0.001

Data are expressed as numbers (%). Analyzed by the Chi-square test for association between genotypes of ABCG2 rs2231142 and hyperuricemia. HUA, hyperuricemia (defined as serum uric acid level>7.0mg/dl); SNP, single nucleotide polymorphisms; OR, odds ratio; 95% CI, 95% confidence interval.

### The Association of BMI With HUA

**Figure 1:**
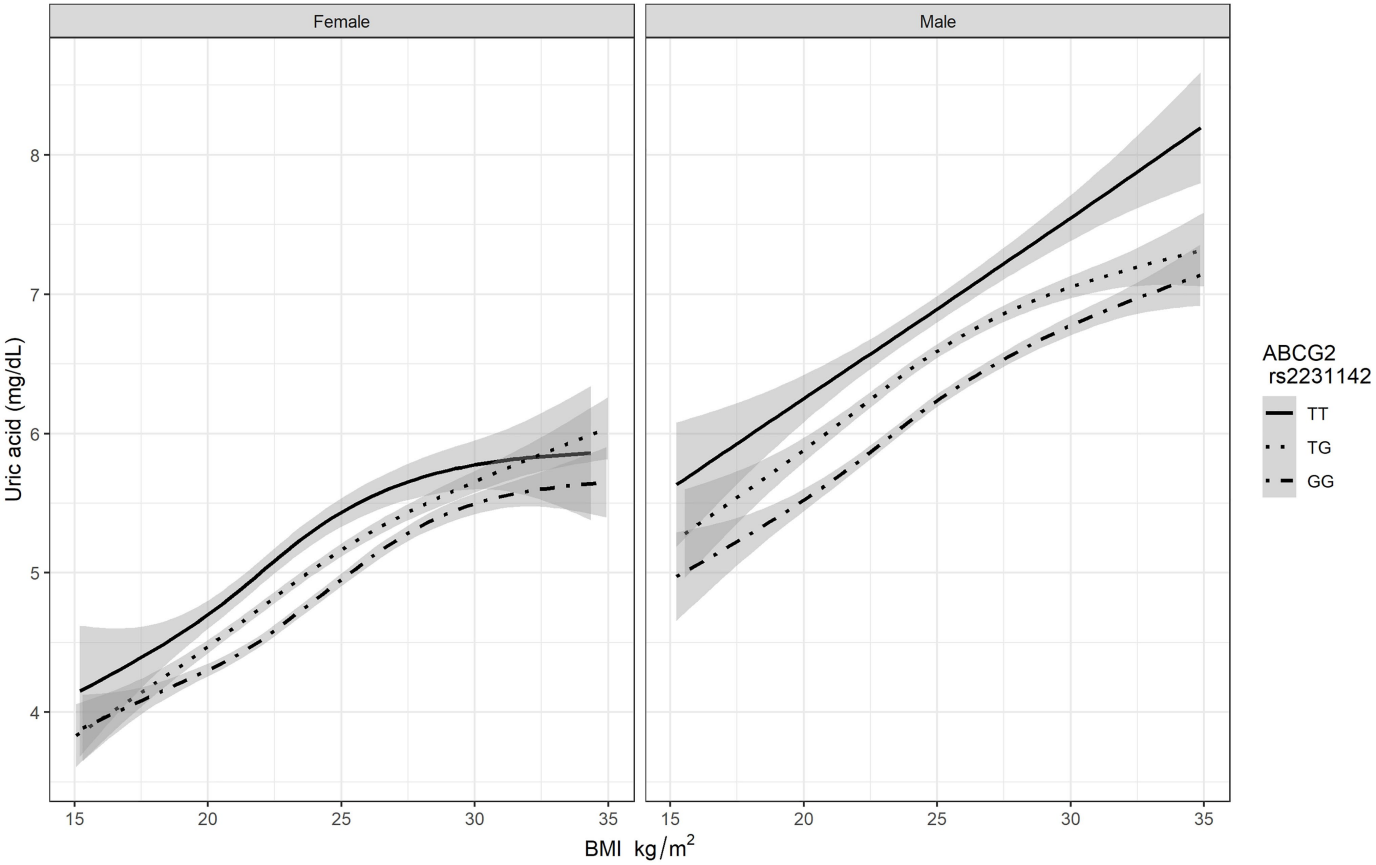
The association BMI and serum uric acid level based on different genotypes of *ABCG2* rs2231142.

After adjusting for potential confounders, BMI was associated with a higher risk of HUA (Table 3). The BMI was analyzed as a continuous variable in the regression analysis, and for each one-unit increase in the BMI, the risk for HUA increased by 11% in men (OR: 1.11, 95% CI: 1.08–1.14, *p*<0.001) and by 15% in women (OR: 1.15, 95% CI: 1.08–1.23, *p*<0.001). In this study, we build a multiple logistic regression model for predicting serum uric acid levels based on *ABCG2* rs2231142 genotypes. As Figure 1 shown, the mean uric acid of people with genotype GG is the lowest, followed by TG, and the highest is of people with TT genotype. As the BMI increased, the serum uric acid level rose in all genotypes, the correlation between BMI and uric acid is positive as a whole. The level of serum uric acid was higher in men than in women, not only at baseline but also at the peak level, and reached a plateau in women with the TT genotype of *ABCG2* rs2231142. In addition, we further characterized the relationship between BMI and serum uric acid levels when BMI exceeds 24 with different genotypes. Based on 3 BMI categories, the distributions of genotypes are almost the same for different gender (Supplementary Figure 1). In female, the mean serum uric acid levels were increased by *ABCG2* rs2231142 risk T allele. Similar results were observed for male where uric acid levels were elevated by TT and TG genotypes increase in BMI, there is an interaction between BMI and *ABCG2* rs2231142 on the effect of uric acid.

**Table 3:** Associations of *ABCG2* rs2231142 and BMI with hyperuricemia.

Gene/SNP	Variables	Male		Female	
		OR (95% CI)	*p* ^a^	OR (95% CI)	*p* ^a^
*ABCG2* rs2231142					
GG		1		1	
TG		1.64 (1.51, 1.78)	<0.001	1.82 (1.46, 2.23)	<0.001
TT		2.40 (2.11,2.72)	<0.001	2.42 (1.83, 3.20)	<0.001
	BMI	1.11 (1.08,1.14)	<0.001	1.15 (1.08,1.23)	<0.001
*ABCG2* rs2231142×BMIs					
GG		1		1	
TG		4.25 (2.13–8.47)	<0.001	3.05 (0.79–11.71)	0.011
TT		7.42 (2.54–21.70)	<0.001	25.43 (3.75–172.41)	<0.001

BMI, body mass index; OR, odds ratio; 95% CI, 95% confidence interval.

a Logistic regression adjusted by age, BMI, hypertension, creatinine, total cholesterol, triglyceride, high density lipoprotein (HDL), and low density lipoprotein (LDL).

### The Effect of Interaction of *ABCG2* rs2231142 and BMI on HUA

*ABCG2* rs2231142 and BMI both had significant impacts on risk of HUA, with genotypes dominant. Table 3 shows the markedly increased risk of HUA due to the interaction of *ABCG2* rs2231142 and BMI. Compared with subjects with the GG genotype of *ABCG2* rs2231142, the risk for HUA increased by 325% (OR: 4.25, 95% CI: 2.13–8.47, *p*<0.001) in men and by 205% (OR: 3.05, 95% CI: 0.79–11.71, *p*=0.011) in women with the TG genotype, and elevated remarkably by 642% (OR: 7.42, 95% CI: 2.54–21.70, *p*<0.001) in men and by 2,443% (OR: 25.43, 95% CI: 3.75–172.41, *p*<0.001) in women with the TT genotype when the BMI increased by one unit.

## Discussion

This cohort study demonstrated a significantly increased chance of HUA in individuals with either the *ABCG2* rs2231142 risk T allele or higher BMI in both men and women. Moreover, we demonstrated a strong genetic-environmental interaction associated with the risk of HUA. Individuals with elevated BMI in combination with the *ABCG2* rs2231142 risk T allele had a much higher risk of HUA compared with the other subgroups. Of note, compared with those without risk factors, the ORs for the TT genotype of *ABCG2* rs2231142 and higher BMI were 7.42 (95% CI 2.54–21.70, *p*<0.001) in men and 25.43 (95% CI 3.75–172.41, *p*<0.001) in women.

We demonstrated that serum uric acid level rose in all genotypes of *ABCG2* rs2231142 as BMI increased, especially for the TT genotype. The gene-environmental interaction between ABCG2 rs2231142 and BMI on the serum uric acid level could be explained by some reasons. The level of ABCG2 and URAT1 increased significantly in an obese mouse model, which might intensify reabsorption of urate (Doshi et al., 2011). Besides, higher BMI and obesity might lead to obesity-induced hyperinsulinemia, enhance renal proximal urate reabsorption and further cause HUA (Facchini et al., 1991; Quiñones Galvan et al., 1995). Our result was similar to a previous study that showed the probability of gout increased when urate genetic risk scores were elevated in different BMI categories (Tai et al., 2020). However, this urate genetic risk score was constructed for gout, not for HUA, and it is unclear whether the score could be completely applicable to HUA prediction. Moreover, the urate genetic score was composed of numerous SNPs, some of which had very low minor allele frequency, and the impact of the top ranking urate loci might be diluted. Additionally, the composite genetic risk score for risk prediction is currently not feasible due to the high cost and the lack of access to this technique. Our study highlighted the effect of *ABCG2* rs2231142, a single first-place genetic variant that impacts serum uric acid in an East Asian population, in order to explore its effects and to identify possible practical measures that could be implemented in clinical practice for treatment and prevention.

In the present study, we found a significantly increased risk of HUA due to the interaction of *ABCG2* rs2231142 and BMI both in men and women. Similarly, Shih-Tsung Cheng and his colleagues determined that the *ABCG2* rs2231142 risk allele exerted a greater effect on frequency of hyperuricemia, especially in obese patients in an East Asian cohort, but the difference in serum uric acid level was not significant in nonobese patients with this risk allele (Cheng et al., 2017). However, the sample size was small, resulting in limited power to detect associations among *ABCG2* rs2231142, BMI, and serum uric acid level in East Asians. Moreover, conflicting results were reported in non-East Asian populations. A study on participants of European descent which analyzed 22 population cohorts (*n*=42,741) showed significant associations of the *ABCG2* rs2231142 variant and BMI with serum uric acid level in males, but not in females (Huffman et al., 2015). Another study revealed no significant effect of interaction between *ABCG2* rs2231142 genotypes and obesity on serum uric acid level in Mexicans (Macias-Kauffer et al., 2019). There were some differences between the results of our study and those reported in previous studies conducted on Western populations, possibly due to ethnic differences, distinct genetic factors, and dietary intake. One meta-analysis demonstrated that the results could be explained by ethnicity, which contributed to 66.67% heterogeneity in the *ABCG2* rs2231142 TT vs. the GG genotype model (Dong et al., 2015). Further subgroup analysis revealed different gout risks in different ethnicities and a similar trend was expected in HUA risk. The odds ratios of gout risk were 2.80 (*p*=0.001), 4.56 (*p*<0.001), and 7.67 (*p*=0.002) for Caucasian, Mongoloid, and Polynesian populations carrying the *ABCG2* rs2231142 TT genotype, respectively, compared with the GG genotype. Apart from genetic variants, dietary habits differed based on distinct ethnicities, and the effects of different diets on obesity status also varied. This has been confirmed in both animal and human studies. C57BL/6 mice fed with a cafeteria diet or a high-fat diet for 12weeks became obese (weight gain 179% on the cafeteria diet and 194% on the high-fat diet), but obesity was not found in those fed a standard chow diet or normal-fat diet (Lang et al., 2019). Since the degree of obesity varied based on different diet habits among ethnicities, and it is well-known that obesity is associated with serum uric acid level according to previous studies (Shirasawa et al., 2020), we might further infer that different dietary habits among ethnicities could be associated with various serum uric acid levels.

There were some strengths in our study. First of all, this study was a population-based study and explored the effects of interaction between *ABCG2* rs2231142 risk allele and BMI on hyperuricemia in a population with a large sample size, which provided enough power for multiple logistic regression analysis and interaction analysis. In addition, the exploration of a single gene effect may be more feasible in clinical practice in the near future. Moreover, the Axiom Genome-Wide TWB Array Plate is a validated chip that is subject to rigorous quality control and is better suited to genotyping of East Asians.

However, this study has some limitations. First, the participants were all East Asians aged 30–70years and thus the results may not be generalizable to Western populations or other age groups (younger than 30years or older than 70years). Further genetics research based on different ethnicities and subgroups are warranted. Second, the majority of subjects recruited from the Taiwan Biobank were healthy, and therefore the results may not be generalizable to hospital-based populations. Third, the BMI of participants was checked at a single time point and may not reflect the trends of BMI change related to the serum level of uric acid. This issue remains to be explored in future studies.

This study sheds new light on the joint effect of *ABCG2* rs2231142 risk allele and BMI on HUA. Based on the results of this study, the authors recommend the following. First, for high-risk patients with the *ABCG2* rs2231142 risk T allele, body weight control and reduction in BMI are recommended to prevent hyperuricemia and possibly avoid development of gouty arthritis as well as associated comorbidities. Second, for high BMI subjects, the *ABCG2* rs2231142 genotype should be checked and the serum uric acid level should also be assessed in those with the *ABCG2* rs2231142 risk T allele. Third, for an individual with elevated BMI, the trend of serum uric acid should be monitored frequently with early implementation of a primary prevention strategy, such as dietary control, weight reduction, and lifestyle modification, in order to prevent development of hyperuricemia.

## Conclusion

We conducted a case–control study using a population-based biomedical research database of subjects with Taiwanese Han Chinese ancestry. The risk of HUA was markedly increased by the interaction of *ABCG2* rs2231142 variant and BMI, both in men and in women. Therefore, body weight control and reduction in BMI are strongly recommended in high-risk patients with the *ABCG2* rs2231142 risk T allele. Prospective studies are needed to evaluate the effects of BMI change on serum uric acid level in individuals with the *ABCG2* rs2231142 risk T allele, which is a high-risk gene.

## Data Availability Statement

The datasets presented in this study can be found in online repositories. The names of the repository/repositories and accession number(s) can be found in the article/Supplementary Material.

## Ethics Statement

The studies involving human participants were reviewed and approved by the Ethics Committee of Taichung Veterans General Hospital’s Institutional Review Board (IRB no. CE16270B-1). The patients/participants provided their written informed consent to participate in this study.

## Author Contributions

C-HL, Y-MC, and Y-JC conceived and designed the study. C-HL, Y-MC, P-CC, Y-JC, Y-CL, and I-CC performed the literature search and interpretation of data. Y-JC drafted the manuscript. P-CC conducted data extraction and methodological quality assessments, and performed the analysis. C-HL, P-CC, Y-MC, T-HH, Y-CL, J-CC, I-CC, and H-JL performed the critical revision of the manuscript for important intellectual content. All authors contributed to the article and approved the submitted version.

## Conflict of Interest

The authors declare that the research was conducted in the absence of any commercial or financial relationships that could be construed as a potential conflict of interest.

## Publisher’s Note

All claims expressed in this article are solely those of the authors and do not necessarily represent those of their affiliated organizations, or those of the publisher, the editors and the reviewers. Any product that may be evaluated in this article, or claim that may be made by its manufacturer, is not guaranteed or endorsed by the publisher.

## Supplementary Material

Supplementary Figure 1 Box plots for the relationship between BMI and serum uric acid levels when BMI exceeds 24 with different genotypes.
