# Supplementary figures and images for: Association of ABCG2 rs2231142 Allele and BMI With Hyperuricemia in an East Asian Population
Source: Front Genet. 2021 Aug 31;12:709887. doi: 10.3389/fgene.2021.709887 (PMC8438144; doi:10.3389/fgene.2021.709887)

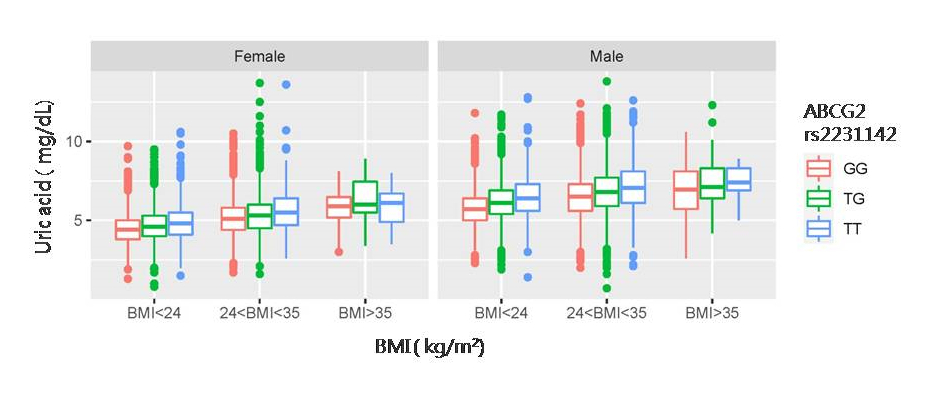

Supplement: Supplementary Figure 1 — Box plots for the relationship between BMI and serum uric acid levels when BMI exceeds 24 with different genotypes. [file Image_1.JPEG]
